# The thiazide sensitive sodium chloride co-transporter NCC is modulated by site-specific ubiquitylation

**DOI:** 10.1038/s41598-017-12819-0

**Published:** 2017-10-11

**Authors:** Lena L. Rosenbaek, Federica Rizzo, Qi Wu, Lorena Rojas-Vega, Gerardo Gamba, Nanna MacAulay, Olivier Staub, Robert A. Fenton

**Affiliations:** 1 InterPrET Center, Department of Biomedicine, Aarhus University, Aarhus, DK-8000 Denmark; 2 Center for Neuroscience, University of Copenhagen, Copenhagen, Denmark; 3 Department of Pharmacology and Toxicology, University of Lausanne, Lausanne, Switzerland; 4 National Centre of Competence in Research “Kidney.ch”, Zurich, Switzerland; 5 Molecular Physiology Unit, Instituto de Investigaciones Biomédicas, Universidad Nacional Autónoma de México and Instituto Nacional de Ciencias Médicas y Nutrición Salvador Zubirán, Mexico City, Mexico

## Abstract

The renal sodium chloride cotransporter, NCC, in the distal convoluted tubule is important for maintaining body Na^+^ and K^+^ homeostasis. Endogenous NCC is highly ubiquitylated, but the role of individual ubiquitylation sites is not established. Here, we assessed the role of 10 ubiquitylation sites for NCC function. Transient transfections of HEK293 cells with human wildtype (WT) NCC or various K to R mutants identified greater membrane abundance for K706R, K828R and K909R mutants. Relative to WT-NCC, stable tetracycline inducible MDCKI cell lines expressing K706R, K828R and K909R mutants had significantly higher total and phosphorylated NCC levels at the apical plasma membrane under basal conditions. Low chloride stimulation increased membrane abundance of all mutants to similar or greater levels than WT-NCC. Under basal conditions K828R and K909R mutants had less ubiquitylated NCC in the plasma membrane, and all mutants displayed reduced NCC ubiquitylation following low chloride stimulation. Thiazide-sensitive sodium-22 uptakes were elevated in the mutants and internalization from the plasma membrane was significantly less than WT-NCC. K909R had increased half-life, whereas chloroquine or MG132 treatment indicated that K706 and K909 play roles in lysosomal and proteasomal NCC degradation, respectively. In conclusion, site-specific ubiquitylation of NCC plays alternative roles for NCC function.

## Introduction

In the mammalian kidney, the sodium chloride cotransporter NCC (*Slc12a3*) of the distal convoluted tubule (DCT) plays a major role in determining body Na^+^ and K^+^ homeostasis^1^. This essential role is emphasized by various diseases in which the activity of NCC is altered, leading to changes in blood pressure and electrolyte balance, e.g. Gitelman or Gordon syndrome^2–6^. NCC activity can be regulated by a variety of hormones, including angiotensin-2 (ANGII)^2,7^, aldosterone^8^ and arginine-vasopressin (AVP)^9^. Although it is intensely debated whether the effects of these hormones are direct or indirect via secondary alterations in serum electrolyte levels^10,11^, they all influence NCC activity to some degree by activating the WNK-SPAK signaling network in DCT cells^6^. Ultimately, activation of these networks results in increased phosphorylation of NCC and higher NCC activity^6^.

Another post-translational modification crucial for regulating various cellular functions, e.g. as a signal for membrane protein endocytosis or degradation of proteins by proteasomes or lysosomes, is ubiquitylation (reviewed in ref.^12^). Recent large-scale proteomics studies have indicated that NCC is ubiquitylated on at least 16 conserved lysine residues^13,14^, but the role of each of these sites for modification of NCC function is unclear. In general, ubiquitylation can target NCC for endoplasmic reticulum-associated degradation (ERAD) by the Hsp70/Hsp40 chaperone system and the C terminus of Hsp70-interacting protein (CHIP)^15,16^. Increased NCC ubiquitylation following phorbol ester treatment^17^ was associated with increased NCC endocytosis, whereas decreased ubiquitylation though a dual-specificity protein phosphatase (DUSP)6-dependent ERK1/2 signal pathway^18^ increased NCC membrane abundance. The ubiquitin-protein ligase NEDD4-2 is implicated in NCC ubiquitylation, as modulation of NEDD4-2 function *in vitro* or *in vivo* modulates NCC abundance and plasma membrane levels^19,20^. The effects of NEDD4-2 on NCC are partially eliminated by WNK3^21^, and although NEDD4-2 co-immunoprecipitates with NCC^19^, whether it directly ubiquitylates NCC is unclear. Finally, greater levels of NCC at the apical plasma membrane are associated with increased phosphorylation of NCC, alongside decreased NCC ubiquitylation and decreased NCC endocytosis^22,23^.

Despite these initial discoveries, the precise roles of individual ubiquitylation sites in NCC were not known. Therefore, in this study 10 different K to R mutations of NCC were generated, expressed in mammalian cell systems and their effect on NCC function examined. Three of the sites (K706, K828, K909) that had greater plasma membrane abundance in HEK cells were systematically evaluated in greater detail in polarized MDCKI cells with respect to their role in modulating total or phosphorylated NCC plasma membrane abundance, NCC internalization rate, NCC protein half-life, NCC activity, and influence on NCC degradation pathways. The results demonstrate that site-specific ubiquitylation of NCC plays alternative roles for modulation of NCC function, with ubiquitylation of NCC at K909 being of major importance for modulating NCC plasma membrane levels and function.

## Results

### Screening of NCC ubiquitin-deficient mutants in HEK293 cells

**Figure 1:**
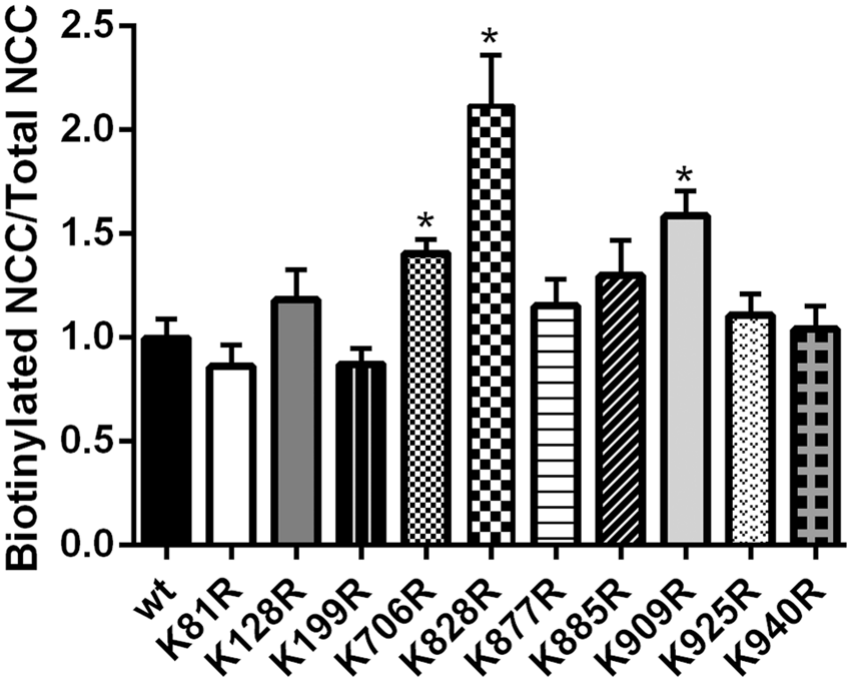
Mutation of 3 different lysine residues within NCC increases membrane abundance of NCC when expressed in HEK293 cells. HEK293 cells were transiently transfected with WT-NCC or 10 different K-R mutants, biotinylated and NCC levels assessed by immunoblotting. Data is semi-quantitative assessment of NCC membrane abundance (biotinylated pool) normalized for the total cellular NCC abundance. Data were analyzed using unpaired Student’s t-test and presented as means ± S.E.M. (*n* = 3–6). *Indicates significant difference compared to WT-NCC.

A Ubiscan screen on mouse kidney lysates identified several lysines within NCC that were ubiquitylated (Table 1). These lysines complemented NCC ubiquitylation sites identified in previous large-scale mass spectrometry studies of mouse kidney^13^ or human urinary exosomes^14^ (Table 1). These data show that endogenous NCC is strongly ubiquitylated on at least 16 different sites. Ten conserved lysines were selected for individual mutational analysis (K to R) within human NCC (hNCC) to evaluate their selective role in NCC function. As we have previously demonstrated that increased apical membrane levels of NCC are associated with decreased NCC ubiquitylation^23^, we focused our initial studies on identifying NCC mutants that had greater plasma membrane abundance. WT-NCC or mutants were transiently transfected into HEK293 cells and cell surface expression of NCC assessed by cell surface biotinylation. Compared to WT-NCC, the K706R, K828R and K909R mutants had significantly higher plasma membrane NCC levels (relative to total NCC) (Fig. 1), suggesting a role of these sites in modulating NCC surface expression.

**Table 1:** Ubiquitylation sites in NCC identified by Ubiscan in this study or from previously published studies^13,14^.

AA position human and (mouse) NCC	Ubiscan of mouse kidney	Human urinary exosomes	Wagner *et al*., 2012	Conserved (mouse and human)	Location	Examined in this study
81 (79)	+		+	+	amino terminus	+
94 (92)		+		+	amino terminus	
128 (126)	+	+	+	+	amino terminus	+
199 (197)			+	+	Intracellular loop 1	+
706 (704)			+	+	carboxyl terminus	+
784 (782)		+		−	carboxyl terminus	
801 (799)		+		−	carboxyl terminus	
814 (795)		+		−	carboxyl terminus	
828 (809)	+		+	+	carboxyl terminus	+
877 (858)			+	+	carboxyl terminus	+
885 (866)	+			+	carboxyl terminus	+
902 (883)	+		+	−	carboxyl terminus	
909 (890)	+	+	+	+	carboxyl terminus	+
925 (906)			+	+	carboxyl terminus	+
940 (921)			+	+	carboxyl terminus	+
948 (929)	+		+	+	carboxyl terminus	

The location of the sites relative to NCC topology and the conserved sites examined further in this study are highlighted.

### Characterization of tetracycline inducible MDCKI-hNCC cell lines

**Figure 2:**
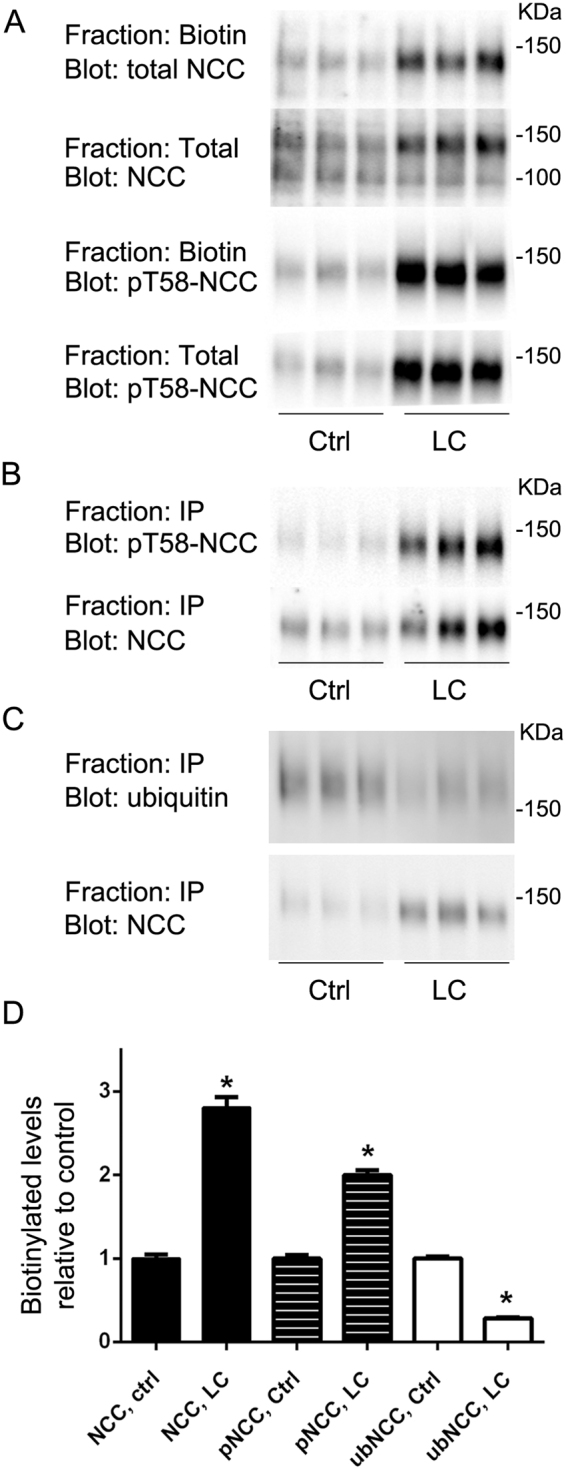
In MDCKI cells, hypotonic low chloride treatment significantly increases levels of total and phosphorylated hNCC at the apical plasma membrane and decreases levels of ubiquitylated hNCC. (**A**) Representative immunoblots of NCC and pT58-NCC in total or biotinylated pools isolated from MDCKI cells under control conditions (Ctrl) or following low chloride (LC) stimulation. (**B**) Representative immunoblots of NCC and pT58-NCC levels in samples immunoprecipitated (anti-FLAG-tag antibody) from the biotinylated pool of MDCKI cells under Ctrl or following LC stimulation. (**C**) Representative immunoblots of NCC and ubiquitylated NCC levels in samples immunoprecipitated (anti-FLAG-tag antibody) from the biotinylated pool of MDCKI cells under Ctr) or following low LC stimulation. (**D**) Semi-quantitative assessment of experiments. Biotin NCC levels are relative to total NCC levels. Biotin pT58-NCC levels are relative to total pT58-NCC levels. Ubiquitylated NCC (ubNCC) levels at the plasma membrane are relative to total NCC levels at the plasma membrane. Data were analyzed using an unpaired Student’s t-test and presented as means ± S.E.M. (*n* = 3–6). *Indicates significant difference compared to corresponding Ctrl conditions.

To study the role of the three hNCC mutants with increased surface expression in detail, we switched to using a polarized MDCKI cell model that allows gene expression under the control of a tetracycline repressor^23^. This system was previously shown to be an excellent system to study endocytosis pathways of rat NCC variants^23^, with hypotonic low chloride treatment of the cells resulting in increased NCC phosphorylation, decreased NCC ubiquitylation and greater levels of NCC at the plasma membrane^23^. Similarly, relative to control conditions, hypotonic low chloride treatment of MDCKI-hNCC WT cells resulted in significantly increased levels of total and phosphorylated NCC at the apical plasma membrane and decreased levels of ubiquitylated NCC (Fig. 2); confirming MDCKI cells as a good model to study hNCC function.

**Figure 3:**
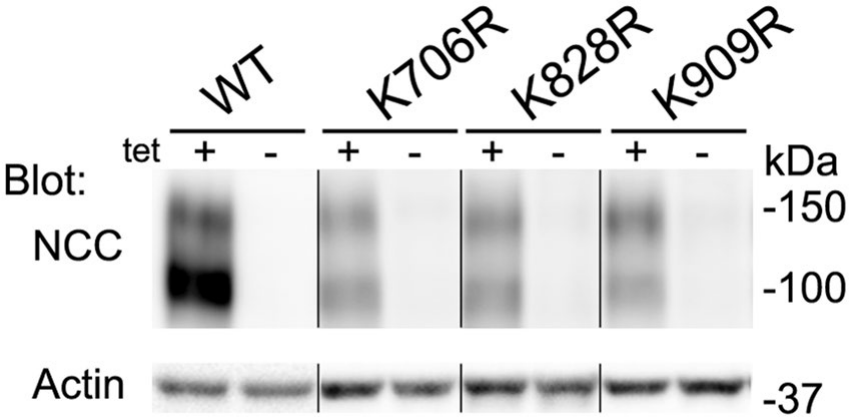
Isogenic MDCKI cell lines for WT-NCC and K706R, K828R and K909R mutants show tetracycline inducible NCC expression. Representative immunoblots of NCC and actin in protein samples isolated from various MDCKI cell lines grown on plastic. Following tetracycline (10 µg/ml) induction NCC is observed as a core non-glycosylated band around 100 kDa and a protein smear at 130 kDa corresponding to the glycosylated monomer. Samples are not run on the same blot, hence a direct comparison of expression levels is not possible.

Several isogenic MDCKI cell lines for K706R, K828R and K909R mutants were generated and initially characterized based on uniform cell morphology, growth characteristics, tetracycline-inducible NCC expression and trafficking of NCC to the apical plasma membrane. All cell lines showed robust NCC expression following 10 μg/ml tetracycline treatment for 16–20 hours, with a core non-glycosylated band around 100 kDa and a protein smear corresponding to the glycosylated monomer centered at 130 kDa (representative blot in Fig. 3). No NCC expression was observed in the absence of tetracycline. One clone for each mutant was selected for the remainder of the study.

### Specific ubiquitylation sites modulate total abundance, phosphorylation levels and degree of NCC ubiquitylation at the plasma membrane

**Figure 4:**
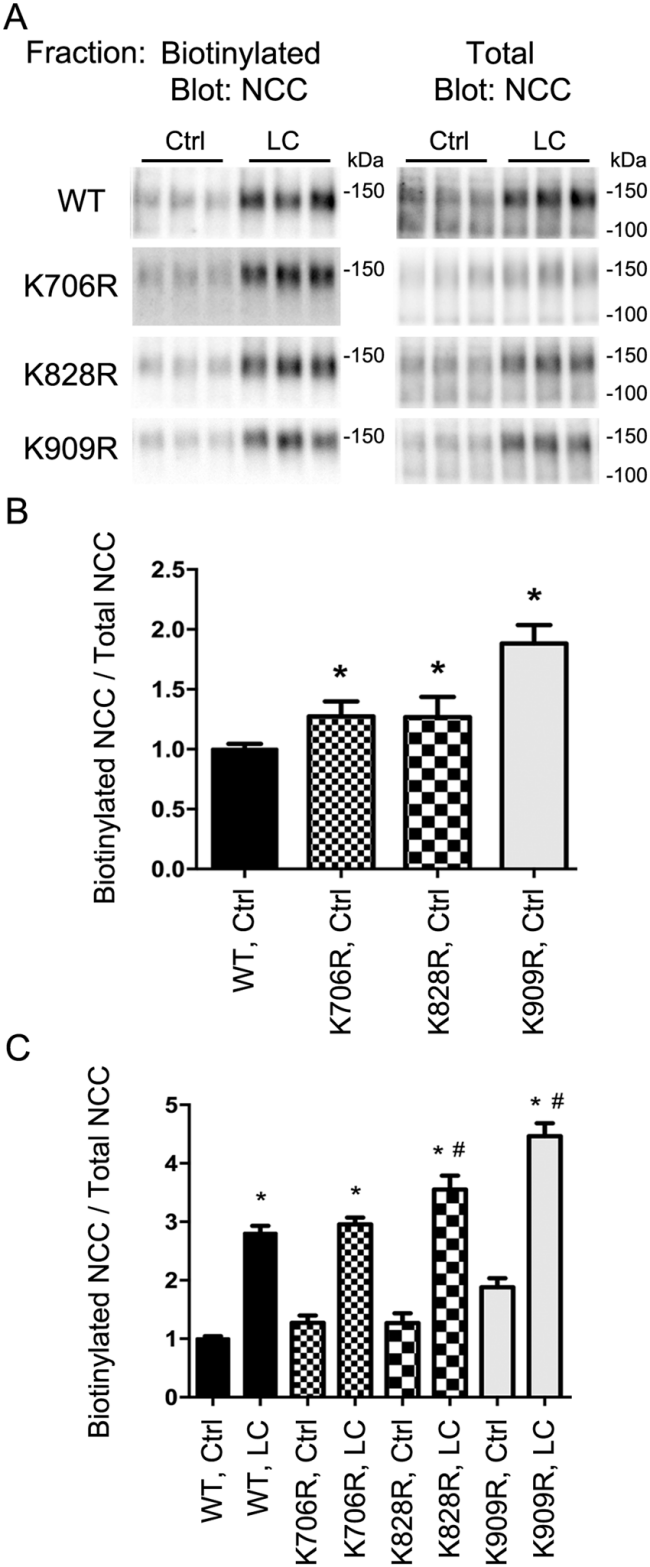
Mutation of K706, K828, and K909 residues in NCC increases membrane abundance of NCC when expressed in MDCKI cells. (**A**) Representative immunoblots of NCC from an apical membrane biotinylation of various MDCKI cells under control (Ctrl) or low chloride (LC) conditions. (**B**) Semi-quantitative assessment of NCC levels at the plasma membrane under Ctrl conditions. Data were analyzed using one-way ANOVA followed by Tukey-Kramer multiple comparison test and presented as means ± S.E.M. (*n* = 6–9) *indicates significant difference compared to WT-NCC under Ctrl conditions. (**C**) Semi-quantitative assessment of NCC levels at the plasma membrane under Ctrl or LC conditions. Data were analyzed using a two-way ANOVA followed by Tukey-Kramer multiple comparison test. Data are means ± S.E.M. (*n* = 6–9) *indicates significant difference between LC and Ctrl conditions for individual cell line. ^#^Represents significant difference compared to WT-NCC following LC stimulation.

**Figure 5:**
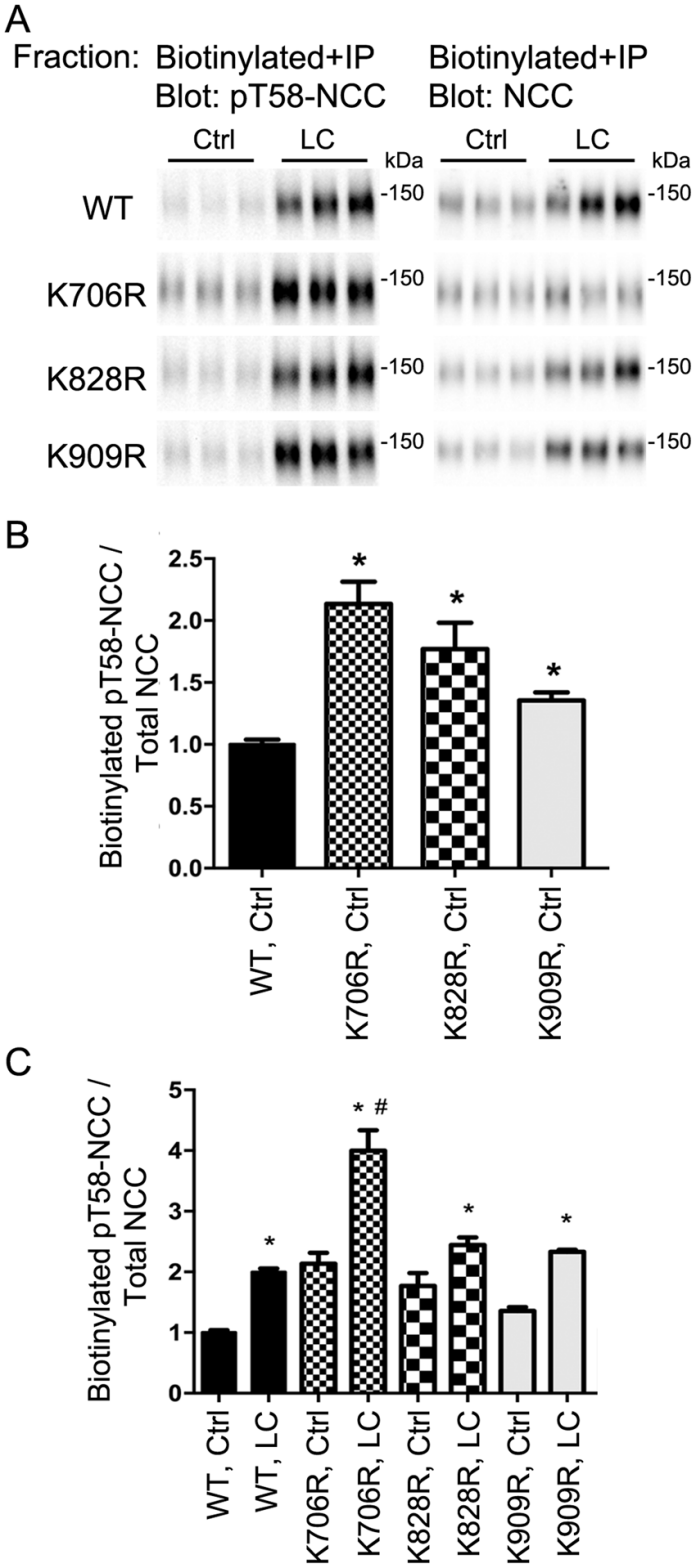
Mutation of K706, K828, and K909 residues in NCC increases membrane abundance of pT58-NCC when expressed in MDCKI cells. (**A**) Representative immunoblots of pT58-NCC and NCC in immunoprecipitated (anti-FLAG-tag) samples isolated from the apical biotinylated pool of various MDCKI cells under control (Ctrl) or low chloride (LC) conditions. (**B**) Semi-quantitative assessment of pT58-NCC levels at the plasma membrane under Ctrl conditions. Data were analyzed using one-way ANOVA followed by Tukey-Kramer multiple comparison test and presented as means ± S.E.M. (*n* = 6–9) *indicates significant difference compared to WT-NCC under Ctrl conditions. (**C**) Semi-quantitative assessment of pT58-NCC levels at the plasma membrane under Ctrl or LC conditions. Data were analyzed using a two-way ANOVA followed by Tukey-Kramer multiple comparison test. Data are means ± S.E.M. (*n* = 6–9) *indicates significant difference between LC and Ctrl conditions for individual cell line. ^#^Represents significant difference compared to WT-NCC following LC stimulation.

**Figure 6:**
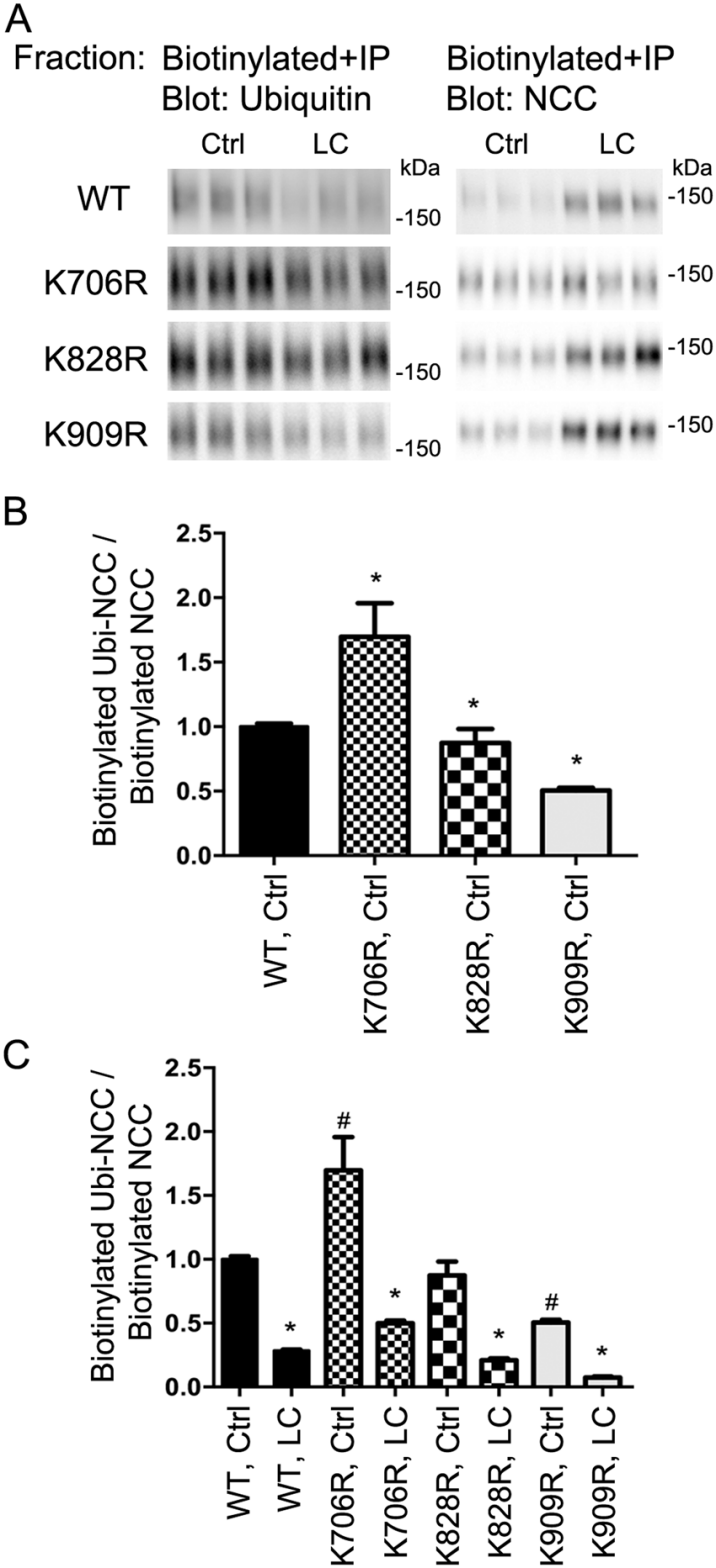
Mutation of K706, K828 and K909 residues in NCC alters apical plasma membrane ubiquitylation levels of NCC when expressed in MDCKI cells. (**A**) Representative immunoblots of ubiquitin and NCC in immunoprecipitated (anti-FLAG-tag) samples isolated from the apical biotinylated pool of various MDCKI cells under control (Ctrl) or low chloride (LC) conditions. (**B**) Semi-quantitative assessment of ubiquitylated NCC (Ubi-NCC) levels at the plasma membrane under Ctrl conditions. Data were analyzed using one-way ANOVA followed by Tukey-Kramer multiple comparison test and presented as means ± S.E.M. (*n* = 6–9) *indicates significant difference compared to WT-NCC under Ctrl conditions. (**C**) Semi-quantitative assessment of ubiquitylated NCC (Ubi-NCC) levels at the plasma membrane under Ctrl or LC conditions. Data were analyzed using a two-way ANOVA followed by Tukey-Kramer multiple comparison test. Data are means ± S.E.M. (*n* = 6–9) *indicates significant difference between LC and Ctrl conditions for individual cell line. ^#^Represents significant difference compared to WT-NCC under ctrl conditions.

To complement the studies in HEK293 cells, apical cell surface biotinylation of the various MDCKI-hNCC cells was performed to assess NCC membrane levels. K706R, K828R, and K909R NCC mutants had a significantly higher membrane abundance compared to WT-NCC (Fig. 4). Following hypotonic low chloride stimulation, plasma membrane levels of all mutants were increased (Fig. 4), with K828R and K909R mutants having significantly greater levels of NCC in the membrane relative to WT-NCC (although the fold changes in response to stimulation relative to WT-NCC are not significantly different). To study phosphorylation and ubiquitylation levels of NCC in the same sample, NCC was immunoprecipitated from the apically biotinylated pool (Fig. 5). All mutants had significantly higher membrane levels of phosphorylated NCC (Fig. 5B) and hypotonic low chloride treatment (Fig. 5C) resulted in greater phosphorylated levels of NCC at the apical plasma membrane relative to WT-NCC, (but comparable fold changes). The total ubiquitylation levels of the K828R and K909R NCC mutants at the plasma membrane were significantly decreased compared to WT-NCC (Fig. 6A and B), whereas surprisingly the levels for K706R were significantly greater. Hypotonic low chloride stimulation reduced membrane-associated NCC ubiquitylation levels in all mutants, with levels of ubiquitylation in K828R and K909R mutants significantly lower than WT-NCC (Fig. 6C). Following hypotonic low chloride treatment, ubiquitylation levels of the K706R NCC mutant remained higher than the corresponding WT-NCC, suggesting that eliminating ubiquitylation at K706 increased ubiquitylation of NCC at another undetermined site.

### Higher NCC activity in NCC ubiquitylation-deficient mutants

**Figure 7:**
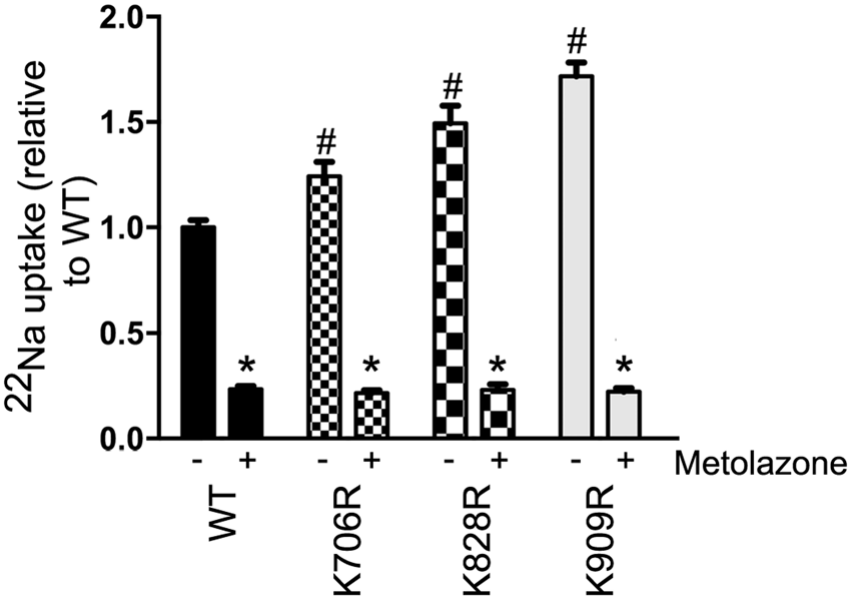
MDCKI cells expressing K706R, K828R and K909R mutants have significantly higher ^22^Na fluxes relative to WT-NCC expressing cells. Quantitative assessment of ^22^Na uptake in various MDCKI cells in the presence (+) or absence (−) of metolazone (Met) in the uptake medium. Data were analyzed using a two-way ANOVA followed by Tukey-Kramer multiple comparison test. Data are means ± S.E.M. (*n* = 12–24). *Represents significant difference compared to without metolazone condition. ^#^Represents significant differences compared to WT-NCC without metolazone.

NCC transport activity correlates with greater NCC membrane abundance and phosphorylation levels^24^. To confirm that the greater membrane levels of the various NCC mutants in the MDCKI cells corresponded with greater NCC transport activity we utilized a newly developed sodium-22 (^22^Na) uptake assay^25^. Cells were grown on filters and following intracellular chloride depletion for 20 min, incubated from the apical side with ^22^Na for 20 min. MDCKI cells expressing K706R, K828R and K909R mutants had significantly higher ^22^Na fluxes relative to WT-NCC expressing cells (Fig. 7). In the presence of the NCC inhibitor metolazone, ^22^Na uptake was significantly reduced to similar levels in all cell lines.

### Ubiquitylation at K706, K828 and K909 plays a role in NCC internalization from the apical plasma membrane

**Figure 8:**
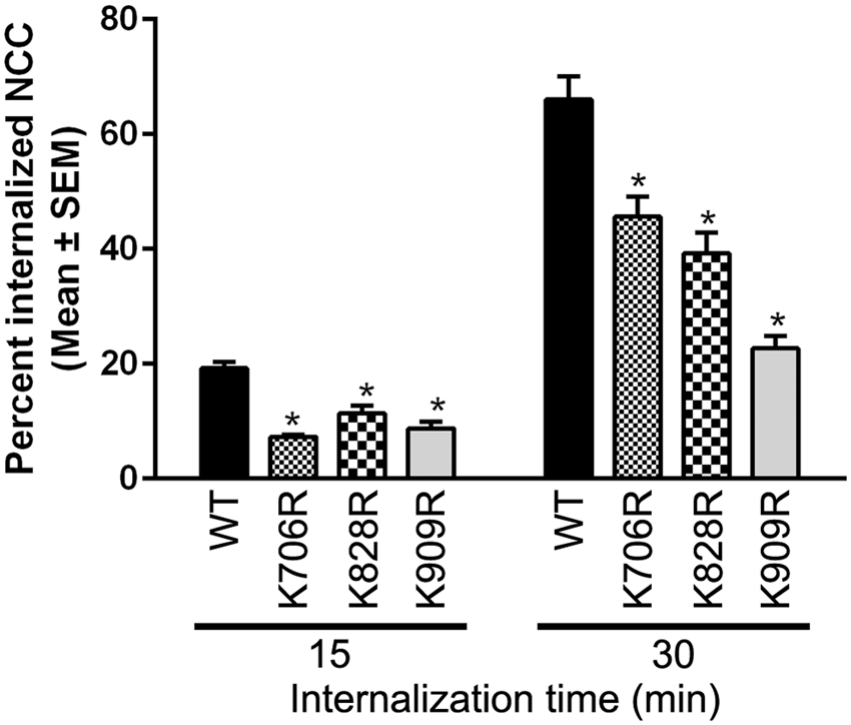
Mutation of K706, K828 and K909 in NCC decreases constitutive internalization of NCC from the apical plasma membrane. Semi-quantitative assessment of the percentage of internalized NCC (steady-state surface levels equals 100%) at various time points. NCC in the total protein fraction (densitometry reading) was initially normalized to Proteasome 20 S to eliminate differences in total protein amount between cells treated with and without MesNa and subsequently NCC in the biotinylated samples was normalized to this value. Data were analyzed using a two-way repeated-measures ANOVA followed by a Holm-Šídák multiple comparison test. Data are means ± S.E.M. (*n* = 6–11). *Represents significant differences compared to WT-NCC at the corresponding time points.

In MDCKI cell lines, previous data indicate that rat NCC was constitutively internalized from the apical plasma membrane; a process decreased by the higher levels of NCC phosphorylation and reduced NCC ubiquitylation subsequent to intracellular chloride depletion^23^. To investigate the role of site-specific ubiquitylation for regulation of NCC endocytosis, biotin-based internalization assays were performed on the MDCKI cells. Relative to WT-NCC, all mutants had significantly decreased constitutive internalization from the plasma membrane after 15 and 30 min (Fig. 8). The degree of internalization was variable between the mutants, with the K909R mutant being the slowest with approximately 50% less internalized NCC after 30 min compared to the time-matched WT-NCC (Fig. 8).

### Ubiquitylation of NCC at specific lysines affects protein half-life and influences lysosomal or proteasomal NCC degradation

**Figure 9:**
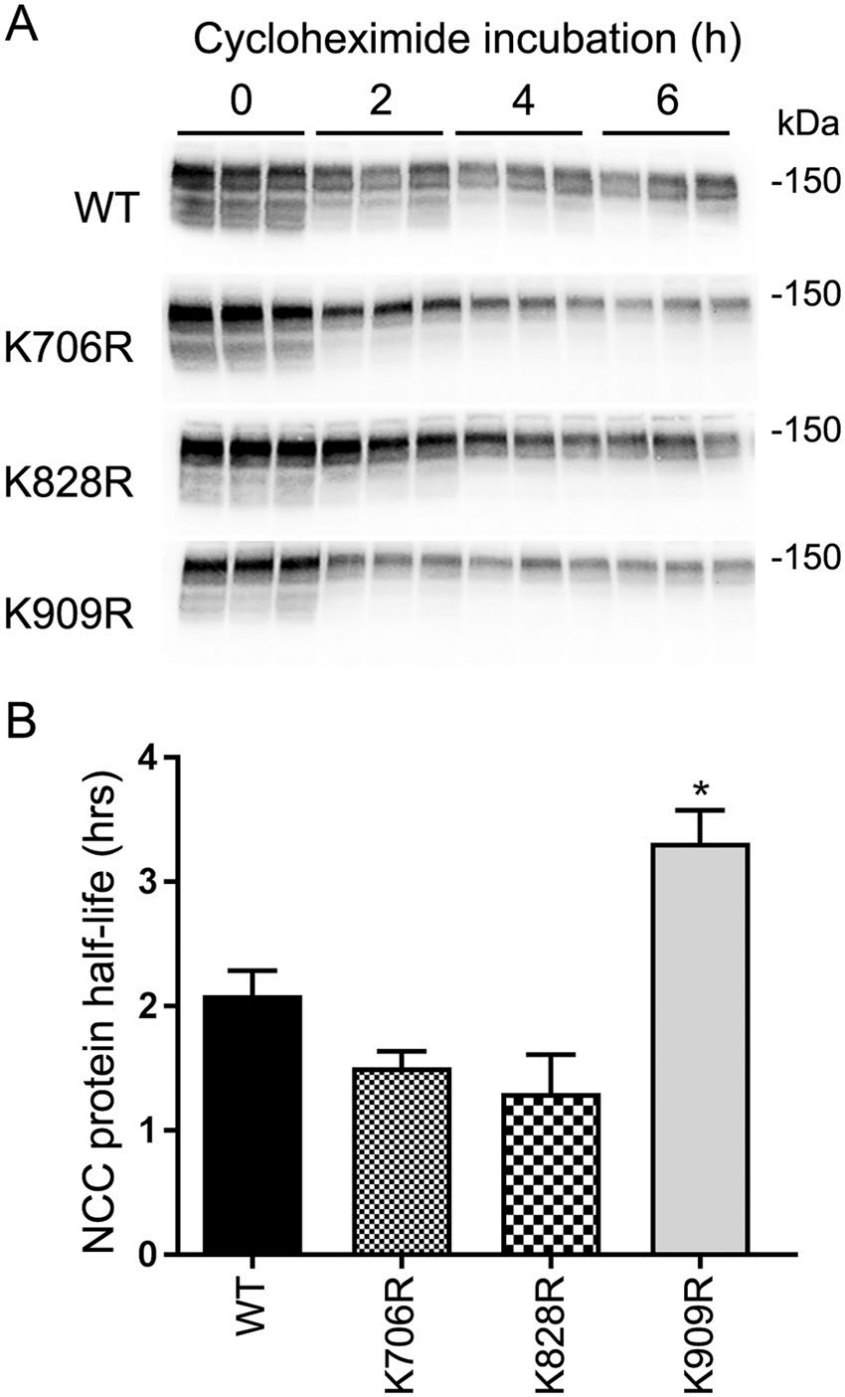
Mutation of K909 in NCC increases the protein half-life of NCC when expressed in MDCKI cells. (**A**) Representative immunoblots of NCC in lysates isolated from various MDCKI cell lines treated with cycloheximide for indicated time. Half-lives of WT and mutants were calculated for each experiment with a one-phase exponential decay analysis. (**B**) Summary of NCC protein half-life from 3 independent analyzed by unpaired Student’s t-test *Represents significant difference compared to WT-NCC.

**Figure 10:**
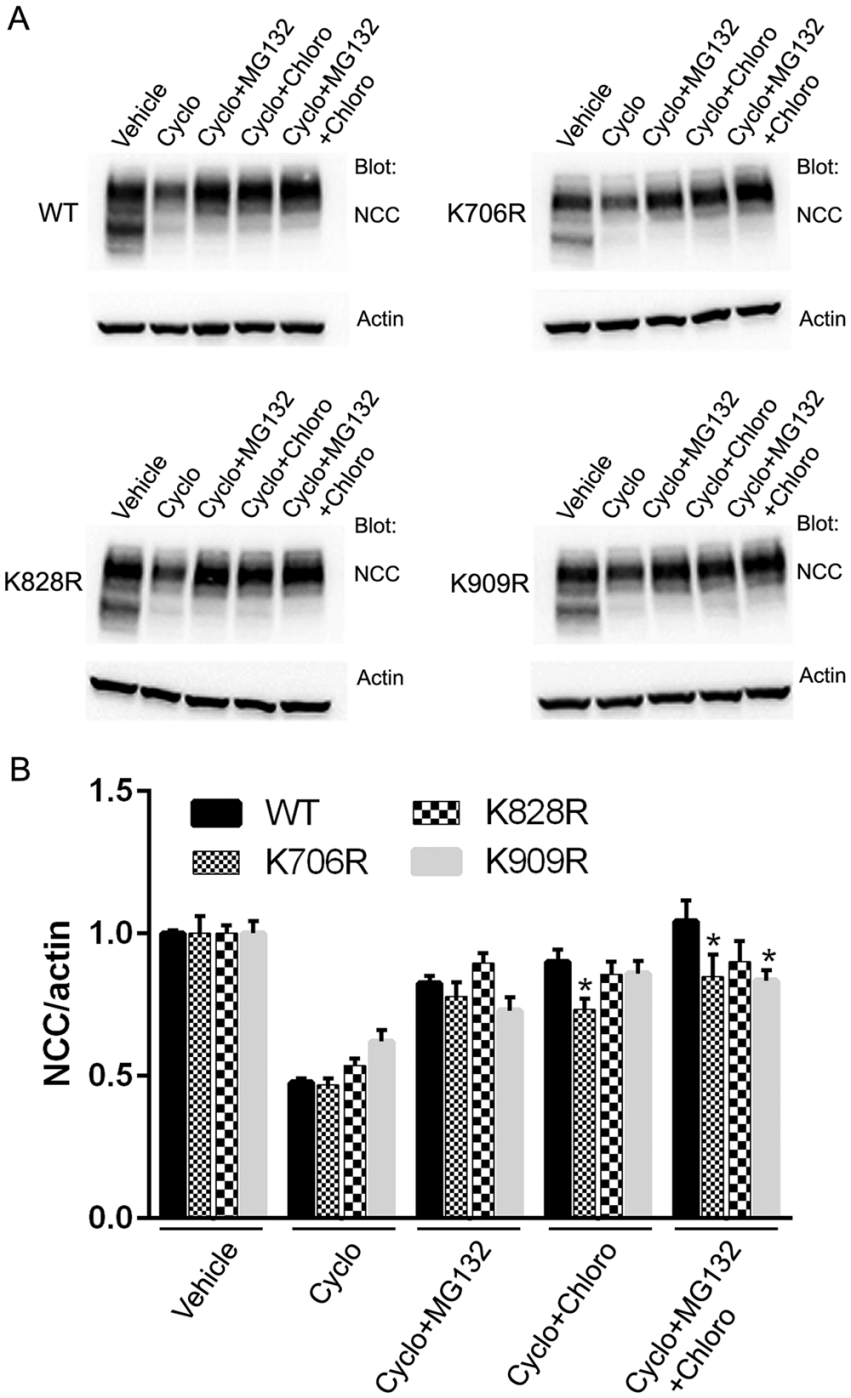
Mutation of NCC at specific lysines influences lysosomal or proteasomal NCC degradation when expressed in MDCKI cells. (**A**) Representative immunoblots of NCC in lysates isolated from various MDCKI cell lines treated with vehicle (ethanol), 50 µM cycloheximide, 50 µM cycloheximide plus 20 µM MG132, 50 µM cycloheximide plus 400 µM chloroquine, 50 µM cycloheximide plus 20 µM MG132 and 400 µM chloroquine for indicated time. (**B**) Semi-quantitative analysis of NCC levels in various MDCKI cell lines following the different treatments. NCC levels in cells expressing K706R and K909R mutants are significantly different that WT-NCC expressing cells. Data were analyzed using two-way ANOVA followed by Tukey-Kramer multiple comparison test and presented as means ± S.E.M. (*n* = 6–9). *Represents significant change compared to WT-NCC under similar conditions.

In addition to acting as a signal for endocytosis, ubiquitin can alter the stability of proteins or selectively target them for degradation by the proteasome or lysosomes. To examine if K706R, K828R or K909R mutations altered NCC degradation rate, cycloheximide studies (inhibitor of protein translation) in the various MDCKI cells were performed and NCC protein levels examined by immunoblotting (Fig. 9A). The average WT-NCC protein half-life, calculated from 3 independent experiments (Fig. 9B), was 2.1 ± 0.19 hrs, which was not significantly different than the NCC half-life in K706R or K828R mutants. In contrast, the K909R mutant had a significantly longer half-life (3.3 ± 0.26), compared to WT-NCC. To investigate the degradation pathways of WT-NCC, MDCKI cells were treated with cycloheximide alone or in combination with the proteasome inhibitor MG132 or the lysosome inhibitor chloroquine. After 4 hrs with cycloheximide, WT-NCC abundance was reduced 50% compared to time matched solvent control (Fig. 10). MG132 or chloroquine restored WT-NCC to 80% or 90% of solvent controls, respectively. In the presence of both inhibitors, WT-NCC levels remained similar to solvent controls, indicating that NCC degradation in MDCKI cells involves both proteasomal and lysosomal pathways. The K828R mutant behaved similarly to WT-NCC (Fig. 10), whereas the levels of the K706R mutant did not recover to the same extent as WT-NCC after treatment with chloroquine or MG132/chloroquine. In contrast, the levels of the K909R mutant tended to be lower after MG132 treatment and were significantly lower in the presence of MG132 plus chloroquine, relative to WT-NCC under similar conditions (Fig. 10).

## Discussion

The thiazide-sensitive NaCl co-transporter NCC is essential for salt reabsorption in the DCT, as emphasized by the use of thiazide diuretics for treatment of non-complicated hypertension. NCC function is tightly linked to multisite phosphorylation of NCC, a process that is dependent on increased activity of the WNK-SPAK signaling network^6^ by the hormones ANGII, aldosterone and AVP^2,7–9^. Ubiquitylation also plays a major role in modulating NCC function; but the majority of studies have focused either on an indirect mechanism regulating NCC where the activity of the WNK-SPAK signaling pathway is modulated by a kelch-like 3 (KLHL3) and Cullin 3 (CUL3) E3 ubiquitin-protein ligase complex dependent-degradation of WNK4^6^, or on a role of the ubiquitin-protein ligase NEDD4-2 for modulating NCC function. Here we describe individual ubiquitylation sites identified within endogenous NCC and focus on determining their functional role.

The combined information obtained from an outsourced, commercial Ubiscan® screen with previous knowledge^13,14^, revealed that endogenous NCC is ubiquitylated on at least 16 different sites (Table 1). Although information on ubiquitylation sites within endogenous renal membrane transporters and channels is now available from proteomic screens^13,14^, our study is among the first to identify numerous ubiquitylation sites within such a class of protein and characterize them functionally. Indeed, we selected 10 ubiquitylation sites (conserved between humans and mice) to study in detail. When expressed in HEK cells, only mutations of NCC at K706, K828, or K909 significantly increased the plasma membrane levels of NCC and were selected for extensive analysis. The role of the other 7 NCC ubiquitylation sites examined in HEK cells, plus the other ubiquitylation sites identified in NCC but not selected for analysis (Table 1) remains unknown. Based on the variety of effects ubiquitylation can have on target proteins^12^, identifying which ubiquitin-protein ligases are responsible for ubiquitylation of NCC at individual sites, and whether NEDD4-2 plays a direct role in ubiquitylating one or more of the identified sites requires extensive further investigation.

The greater plasma membrane abundance, combined with reduced internalization rate of the studied mutants, when expressed in MDCKI cells, strengthens the current concept that NCC ubiquitylation, in part, is important for modulating NCC endocytosis^17,18,22,23^. What remains unclear is whether the K706, K828, and K909 sites act alone or together in modulating NCC membrane levels. Technical limitations of the LC-MS/MS based approaches to identify NCC ubiquitylation sites (Table 1), means that it is not known whether an NCC monomer is ubiquitylated at multiple sites simultaneously, or the modification is added differentially, dependent on the cellular localization of NCC. The former possibility is supported by the IP data (Fig. 6), demonstrating that removal of a single ubiquitylation site within NCC does not completely remove ubiquitin from NCC. However, attempts to study NCC in MDCK cells after mutation of 2 or 3 NCC ubiquitylation sites simultaneously were unsuccessful due to severely reduced NCC levels under these conditions (data not shown).

As eluded to earlier, the levels of NCC phosphorylation and ubiquitylation appear to be linked, at least in respect to their contrasting roles in modulating plasma membrane levels of NCC^23^. Here, we saw similar effects on human NCC; significantly greater plasma membrane levels of NCC were associated with decreased plasma membrane levels of ubiquitylated NCC and increased levels of phosphorylated NCC. Consistently, the tested NCC mutants with greater membrane abundance had significantly higher membrane levels of phosphorylated NCC, and all mutants had greater phosphorylated NCC levels following low chloride stimulation alongside significantly higher ^22^Na fluxes relative to WT-NCC expressing cells. Taken together, this data strengthens the idea that NCC phosphorylation and site-specific ubiquitylation are opposing processes that modulate the plasma membrane levels and activity of NCC – but how does this occur? It is well established that there is functional interplay between phosphorylation and ubiquitylation^26^, yet the majority of studies have focused on their interplay to modulate kinase signaling rather than unraveling the mechanisms by which the two modifications on the same protein may impact on membrane protein trafficking^27^. We previously demonstrated that phosphorylation and ubiquitylation are opposing processes that regulate endocytosis of the water channel aquaporin-2 (AQP2), but the levels of the two modifications are mutually exclusive^28^. Here, reduced NCC ubiquitylation corresponded with increased NCC phosphorylation, except for the K706R mutant. As biotinylated NCC can be ubiquitylated, it is likely that a mechanism exists where phosphorylation of NCC within the plasma membrane prevents ubiquitylation or promotes rapid deubiquitylation of NCC. Several scenarios are imaginable. Phosphorylation of NCC may influence directly or indirectly the interaction of ubiquitin-protein ligases or deubiquitylases with NCC. Alternatively, phosphorylation and ubiquitylation of NCC may occur in specific “microdomains” within the plasma membrane, and phosphorylation of NCC may inhibit the movement of NCC to a domain where NCC ubiquitylation and subsequent clathrin-mediated endocytosis can occur. Phosphorylation-dependent redistribution of NCC to different domains of the apical plasma membrane of DCT cells has been proposed^29^, but further experiments are required to define the underlying mechanism. In respect to cells expressing the K706R mutant, although total NCC ubiquitylation levels were increased, phosphorylation was also increased alongside increased ^22^Na uptake. We can only speculate that the increased ubiquitylation occurs at a site that is not influenced by phosphorylation.

Previous studies have suggested that NCC can be degraded by the proteasomal pathway via ERAD^15,16^ and the endosomal/lysosomal pathway^14,30–32^. This concept is supported by the current study where both MG132 and chloroquine limited NCC degradation. Furthermore, the data presented here indicate that site-selective ubiquitylation of NCC likely plays different roles in determining the degradation fate of the cotransporter. Mutation at K909 significantly increased NCC half-life and rendered NCC less sensitive to proteasomal inhibition, suggesting that native K909 ubiquitylation (besides its role in endocytosis) is involved in the degradation of NCC by the proteasomal pathway. In contrast, mutation at K706 rendered NCC less sensitive to chloroquine treatment, suggesting that the prevention of ubiquitylation at lysine 706 reduces the contribution of the lysosomes to NCC degradation. The mechanisms controlling these events are unknown, but as different forms of ubiquitylation (mono-, polyubiquitylation via K48- or K63-linkages) are able to target proteins either to proteasomal or lysosomal pathways^33^, examination of whether NCC has a specific type of ubiquitin chain at these sites using LC-MS/MS based techniques^34^ would be informative.

A question that remains from the current studies is “How much of the total pool of NCC is ubiquitylated”? This is a very difficult question to answer. On immunoblots, the ubiquitylated forms of NCC are not clearly identified at similar exposure times to the non-ubiquitylated forms of NCC. This phenomenon, where the ubiquitylated species cannot be detected with the anti-substrate antibody is not uncommon, as in general the ubiquitylated protein pool at any specific time likely represents a small pool of the total protein for any given target. However, as NCC could be rapidly ubiquitylated and deubiquitylated during the process of endocytosis, even though the absolute pool of ubiquitylated NCC relative to total NCC is small, ubiquitylation of NCC could be of major importance for NCC function. Quantitatively assessing the fraction of NCC that is ubiquitylated is very difficult using antibody-based techniques due to the relative differences in antibody affinities for their substrates. Furthermore, we have no idea of the type of ubiquitin modification of NCC e.g. mono- or poly-ubiquitylation, or whether multiple sites on NCC are ubiquitylated at the same time. The use of Protein AQUA technologies coupled with mass spectrometry^14,35^ to identify the absolute amount, and the type of ubiquitin linkages in NCC could be informative.

In conclusion, we have shown that NCC is highly ubiquitylated *in vivo* on specific sites. We further demonstrated that modification of such sites plays alternative roles in modulating NCC endocytosis and degradation. Future studies examining whether ubiquitylation of NCC is regulated under various physiological conditions, in a similar manner to NCC phosphorylation, would shed new light on regulatory mechanisms controlling DCT NaCl transport and blood pressure.

## Methods

### UbiScan assay of mouse kidney

Experimental protocols were designed with respect to the Swiss Animal Welfare Act and approved by the veterinary administration of the Canton de Vaud (Switzerland). Mice had free access to standard rodent chow and water. C57BL/6 mice were killed by cervical dislocation, the kidneys removed, rinsed with cold PBS and frozen immediately in liquid nitrogen. Ubiscan® ubiquitylation analysis of the kidneys was performed as a commercial PTMScan® Discovery Proteomics service^36^ by Cell Signaling Technology (CST) using proprietary techniques.

### Transient transfection of HEK293 cells and biotinylation assay

A FLAG-tag (amino acids DYKDDDDK, ntd: GACTACAAGGACGATGACGATAAG) was added by PCR at the NH2-terminus of human NCC (WT-NCC) cDNA in pCMV5 vector (the cDNA corresponds to gi|186910319|ref| NP_001119580.1| solute carrier family 12 member 3 isoform 3). Mutations of selected lysines to arginines (K81R, K128R, K199R, K706R, K828R, K877R, K885R, K909R, K925R, K940R) were performed by site directed mutagenesis using standard protocols. WT-NCC and mutants were transiently transfected into HEK293 cells using Lipofectamine® 2000 (Thermo Scientific) using manufacturers’ guidelines. Cells were maintained in DMEM high glucose supplemented with 10% fetal bovine serum, penicillin 5 U/ml and streptomycin 5 µg/ml (all Thermo Scientific). 24 hrs after transfection, biotinylation of surface proteins was performed. Cells were washed twice in ice-cold PBS-CM (PBS, 1 mM CaCl_2_, 0.1 mM MgCl_2_, pH 8.9) and then incubated for 45 min in ice-cold PBS-CM pH 8.9 containing 1.5 mg/ml of sulfosuccinimidyl 2-(biotin-amido)-ethyl-1,3-dithiopropionate (EZ-link Sulfo-NHS-SS-biotin, Pierce) at 4 °C with mild agitation. Cells were incubated in quenching solution (100 mM Glycine in PBS-CM, pH 7.5) twice for 10 min, washed in PBS-CM pH 7.5 and then scraped and lysed in lysis buffer (20 mM Tris–HCl pH 8.0, 150 mM NaCl, 5 mM EDTA, 1% Triton X-100, 0.2% BSA, plus protease inhibitors cocktail (Roche)). Samples were sonicated and centrifugated at 13,000 × g for 10 min at 4 °C. One fraction of the supernatant was retained for total NCC protein estimation (total fraction) and the remaining was incubated overnight with 30 µl of Streptavidin Sepharose High Performance (GE Healthcare) under rotation at 4 °C. Sepharose beads were washed three times in lysis buffer, resuspended in 30 µl of Laemmli sample buffer with 100 mM DTT, and proteins eluted via heating at 42 °C for 30 min followed by centrifugation.

### Tetracycline-inducible MDCKI-hNCC cell lines

The MDCKI-hNCC cell line was generated and partially characterized previously^25^. K706R, K828R and K909R were introduced in the pcDNA5/FRT/TO/TOPO-hNCC WT vector by site directed mutagenesis (Mutagenex, Georgia, USA). Plasmid cDNA vectors were cotransfected using Lipofectamine 2000 (Thermo Scientific) with pOG44 (encoding flp recombinase) into a tetracycline inducible MDCK type I cell line containing a single FRT site in its genome^23^. Positive clones were selected using 500 μg/ml Hygromycin B and stable MDCKI-hNCC cell lines were maintained in DMEM High Glucose with 10% DBS, 150 μg/ml Hygromycin B, and 5 μg/ml Blasticidin HCl (all Thermo Scientific).

### Cell surface biotinylation coupled with immunoprecipitation (IP)

Cells were grown in DMEM High Glucose with 10% DBS on filter plates coated with basement membrane extract (BME) (Cultrex® Basement Membrane Extract, PathClear, R&D Systems) until confluent. Cells were induced with 10 μg/ml tetracycline HCl for 16–20 hrs prior to experiment. Cells were washed twice in isotonic buffer (135 mM NaCl, 5 mM KCl, 1 mM CaCl_2_, 1 mM MgCl_2_, 1 mM Na_2_HPO_4_, 1 mM Na_2_SO_4_, 15 mM Sodium HEPES, pH 7.4) before stimulation, where indicated, with hypotonic low chloride buffer (67.5 mM sodium gluconate, 2.5 mM potassium gluconate, 0.5 mM CaCl_2_, 0.5 mM MgCl_2_, 1 mM Na_2_HPO_4_, 1 mM Na_2_SO_4_, 7.5 mM sodium HEPES, pH 7.4) for 20 min at 37 °C. Cells were subsequently washed in ice-cold PBS-CM (pH 7.5) and incubated with mild agitation for 30 min at 4 °C in ice-cold biotinylation buffer (10 mM triethanolamine, 2 mM CaCl_2_, 125 mM NaCl, pH 8.9) containing 1 mg/ml sulfosuccinimidyl 2-(biotinamido)-ethyl-1,3-dithiopropionate (EZ-link Sulfo-NHS-SS-biotin, Pierce) added to the apical compartment. Cells were washed in ice-cold quenching buffer (PBS-CM containing 50 mM Tris-HCl, pH 8) followed by two washes in PBS-CM. Cells were scraped and sonicated in lysis buffer (20 mM Tris, 150 mM NaCl, 1% Nonidet P-40, 5 mM EDTA (pH 7.4), 20 mM N-ethylmaleimide (Sigma), 22 μM PR619 (Abcam), 5 μg/ml leupeptin, 100 μg/ml Pefabloc, and PhosSTOP phosphatase inhibitor tablets (all from Roche Diagnostics). Samples were centrifuged at 10,000x g for 5 min at 4 °C. One fraction of the supernatant was retained for total NCC protein estimation (total fraction) or subsequent IP. The remaining sample was incubated for 1 hr in spin columns containing NeutrAvidin gel slurry (Pierce) with rotation at 4°C. After extensive washing in lysis buffer, biotinylated proteins were eluted with 50 mM DTT in lysis buffer for 1 hr with rotation at room temperature. A fraction of the biotinylated proteins was retained for surface protein estimates. The remaining sample, alongside the retained total sample, was diluted to 500 μl using lysis buffer and subjected to IP using 2 μg of rabbit FLAG antibody and 20 μl of protein A-agarose (Santa Cruz Biotechnology) at 4 °C overnight with rotation. Resin was washed 3x with lysis buffer and eluted with 200 μg/ml FLAG peptide solution (Genscript) in TBS (10 mM Tris-HCl, 150 mM NaCl, pH 7.4). Laemmli buffer was added (final DTT concentration of 100 mM) and samples were heated for 15 min at 60 °C. Specificity of the IP was confirmed using antibody-deficient samples (Supplemental Fig. 1).

### Antibodies

Rabbit polyclonal antibodies against total NCC^37^ (from Dr. Mark Knepper, NIH, Bethesda, Maryland, USA) or ref.^38^ (from Dr. Johannes Loffing, Institute of Anatomy, University of Zurich, Switzerland), phosphorylated Threonine-58 NCC (pT58)^9^, FLAG-tag (F7425, Sigma), Actin (A2228, Sigma), Proteasome 20S (ab3325), and a mouse monoclonal antibody against ubiquitin (P4DI, Cell Signaling) were used for immunoblotting. Specificity of pT58 NCC antibody was confirmed by lack of signal in NCC knockout mouse kidneys (not shown), ablation of immunohistochemcial labeling of the mouse DCT using peptides containing pT58 but not non-phosphorylated peptides or pT53 peptides^9^, or by blotting of various MDCKI-rNCC cell lines with arginine/aspartic acid mutations at T58 (Supplemental Fig. 2).

### Immunoblotting

Standard procedures were utilized for sample preparation and SDS-PAGE using 4–15% gradient polyacrylamide gels (Criterion TGX Precast Protein Gels, BioRad). Immunoblots were developed using SuperSignal West Femto chemiluminescent substrate (Thermo Scientific, Denmark) or Amersham ECL Western Blotting Detection Reagent (GE Healthcare) detection and signal intensity in specific bands were quantified using Image Studio Lite (Qiagen) densitometry analysis. To facilitate comparisons within the groups for the surface expression experiments (Figs 4–6), all biotinylated samples (per individual experiment) isolated from wildtype and mutant cells are run simultaneously on the same blot (24 samples) and the exposure times are equivalent. A similar approach is used for the total samples and biotin-immunoprecipitation samples from the same experiment. As such, for the wildtype and mutants the numerator and denominator for each normalization step have similar exposures and are thus comparable. The values are normalized to the wildtype within the individual experiment and the results of several individual experiments combined to obtain the final data.

### ^22^Na uptake assay

Performed as previously described^25^. Briefly, various MDCKI-hNCC cells were grown in 12-well plastic culture plates in DMEM High Glucose with 10% DBS to confluency. Cells were induced for 16–20 hrs with 10 μg/ml tetracycline HCl. Cells were washed in pre-heated (37 °C) serum-free DMEM medium and incubated for 20 min at 37 °C in chloride-free buffer (130 mM Na gluconate, 2 mM K gluconate, 1 mM Ca gluconate, 1 mM Mg gluconate, 5 mM HEPES, and 5 mM Tris-HCl, pH 7.4) containing 1 mM ouabain, 1 mM amiloride, 0.1 mM benzamil, and 0.1 mM bumetanide, with or without 0.1 mM metolazone (all Sigma). Cells were incubated in uptake buffer (140 mM NaCl, 1 mM CaCl_2_, 1 mM MgCl_2_, 5 mM HEPES, and 5 mM Tris pH 7.4 including inhibitors) with 1.5 µCi/ml ^22^NaCl (PerkinElmer) for 20 min at 37 °C. Cells were rapidly and extensively washed in ice cold uptake buffer without radioisotope and lysed in 500 µl of PBS-CM with 0.1% SDS. Lysates were counted in a Cobra II 5002 Auto-Gamma counter (Packard). 20 µl of each lysed sample was used to determine total protein concentration using the BCA Protein Assay Kit (Pierce).

### Biotin-based internalization assay

Performed as previously described^23^. Briefly, tetracycline-induced confluent MDCKI cells grown on filter plates were washed twice at room temperature in pure media (without serum) and once in ice-cold PBS/CM (PBS, 1 mM CaCl_2_, 0.1 mM MgCl_2_, pH 7.5). To label apical surface NCC, the apical compartment of cells was incubated for 45 min at 4 °C in ice-cold biotinylation buffer (10 mM triethanolamine, 2 mM CaCl_2_, 125 mM NaCl, pH 8.9) containing 1.5 mg/ml EZ-link Sulfo-NHS-SS-biotin (Pierce). Cells were washed once in ice-cold quenching buffer (PBS-CM, 50 mM Tris-HCl, pH 8) followed by two washes in PBS-CM. At this point NCC surface expression controls (maximum NCC at the cell surface) were made following the standard biotinylation protocol. The remaining cells were incubated in pure media for 0, 15 or 30 min at 37 °C to allow plasma membrane proteins (including NCC) to internalize. At each time point, endocytosis was stopped by rapidly cooling the cells using ice-cold PBS-CM and biotin was stripped from the remaining surface proteins by 3 × 20 min incubations at 4 °C with the membrane-impermeable reducing agent sodium-MES (MesNa, 200 mM) in (100 mM NaCl, 1 mM EDTA, 50 mM Tris, pH 8.6, 0.2% BSA). Reactions were quenched with 120 mM iodoacetic acid in PBS-CM followed by PBS-CM washes. Cells were lysed and biotinylated proteins isolated according to the surface biotinylation protocol. Biotinylated and total protein fractions were analyzed for NCC and proteasome 20 S abundance by immunoblotting. NCC in the total protein fraction (densitometry reading) was initially normalized to Proteasome 20 S to eliminate differences in total protein amount between cells treated with and without MesNa and subsequently NCC in the biotinylated samples was normalized to this value. The stripped sample signal density at the 0-min internalization time point for individual experiments was utilized as background signal for all other time points. The quantity of internalized NCC at each time point is relative to the maximum NCC at the cell surface within an individual experiment.

### NCC protein half-life assay

Various MDCKI-hNCC cells were grown on filter plates to confluency in DMEM High glucose with 10% DBS. Cells were induced with 10 μg/ml tetracycline HCl for 16–20 hrs prior to experiment. Cells were washed once with DMEM and then incubated in 50 µM cycloheximide in DMEM for various times. Cells were washed in PBS-CM pH 7.5 and lysed in Laemmli buffer containing protease inhibitors cocktail (Roche) and DTT (final concentration of 100 mM). Samples were sonicated, heated at 60 °C for 15 min and subjected to immunoblotting. For calculation of the protein half-life, average band densities for each time point were normalized to time zero and fitted using nonlinear regression and a one-phase exponential decay equation using GraphPad Prism software. Data were obtained from three independent experiments, with 3 observations for each individual time point.

### Lysosomal and proteasomal inhibition assays

Various MDCKI-hNCC cells were grown on filter plates to confluency in DMEM High Glucose with 10% DBS. Cells were induced with 10 µg/ml tetracycline HCl for 16–20 hrs prior to experiment. Cells were incubated for 4 hrs in DMEM High Glucose with 10% DBS containing either EtOH (vehicle of cycloheximide), 50 µM cycloheximide, or 50 µM cycloheximide in combination with 20 µM of MG132 (proteasome inhibitor), 400 µM chloroquine (lysosome inhibitor) or both. Cells were washed in PBS-CM and processed as for the half-life assay.

### Data and Statistics

For immunoblotting and uptake assays, data are expressed as mean ± S.E.M. For two groups, data meeting the statistical assumptions of normality were assessed using an unpaired Student’s t-test using Graphpad Prism. Comparisons of more than two groups were performed using either a one-way ANOVA or two-way repeated-measures ANOVA followed by Tukey-Kramer or Holm-Šídák multiple comparison tests, respectively. Experimental numbers (n) are reported in individual figure legends. Significance was considered at P < 0.05.

### Data availability

All materials, data and associated protocols are available to others upon request. No datasets were generated or analyzedduring the current study. We confirm that all methods were performed in accordance with relevant guidelines and regulations.

## Electronic supplementary material

Supplemental Figures and Legends
