# Author Correction: The thiazide sensitive sodium chloride co-transporter NCC is modulated by site-specific ubiquitylation

**DOI:** 10.1038/s41598-018-22153-8

**Published:** 2018-03-06

**Authors:** Lena L. Rosenbaek, Federica Rizzo, Qi Wu, Lorena Rojas-Vega, Gerardo Gamba, Nanna MacAulay, Olivier Staub, Robert A. Fenton

**Affiliations:** 1 InterPrET Center, Department of Biomedicine, Aarhus University, Aarhus, DK-8000 Denmark; 2 Center for Neuroscience, University of Copenhagen, Copenhagen, Denmark; 3 Department of Pharmacology and Toxicology, University of Lausanne, Lausanne, Switzerland; 4 National Centre of Competence in Research “Kidney.ch”, Zurich, Switzerland; 5 Molecular Physiology Unit, Instituto de Investigaciones Biomédicas, Universidad Nacional Autónoma de México and Instituto Nacional de Ciencias Médicas y Nutrición Salvador Zubirán, Mexico City, Mexico

Correction to: *Scientific Reports* 10.1038/s41598-017-12819-0, published online 11 October 2017

In the original version of this Article, the affiliation for Lorena Rojas-Vega and Gerardo Gamba was incomplete. The correct affiliation for these authors appears below.

Molecular Physiology Unit, Instituto de Investigaciones Biomédicas, Universidad Nacional Autónoma de México and Instituto Nacional de Ciencias Médicas y Nutrición Salvador Zubirán, Mexico City, Mexico.

This error has now been corrected in the PDF and HTML versions of the Article.

