# Supplementary material for: The thiazide sensitive sodium chloride co-transporter NCC is modulated by site-specific ubiquitylation
Source: Sci Rep. 2017 Oct 11;7:12981. doi: 10.1038/s41598-017-12819-0 (PMC5636807; doi:10.1038/s41598-017-12819-0)

Supplementary Information

The thiazide sensitive sodium chloride co-transporter NCC is modulated by site-specific ubiquitylation

Lena L Rosenbaek[1,2,§], Federica Rizzo[3,4,§], Qi Wu[1], Lorena Rojas-Vega[5], Gerardo Gamba[5], Nanna MacAulay[2], Olivier Staub[3,4] and Robert A. Fenton[1]

[1]InterPrET Center, Department of Biomedicine, Aarhus University, Aarhus DK-8000, Denmark, [2]Center for Neuroscience, University of Copenhagen, Copenhagen, Denmark, [3]Department of Pharmacology, Lausanne University, Lausanne, Switzerland, [4]National Centre of Competence in Research "Kidney.ch", Switzerland, [5]Molecular Physiology Unit, Instituto de Investigaciones Biomédicas, Universidad Nacional Autónoma de México, Mexico City, Mexico

Supplemental Figure 1. Specificity of the NCC immunoprecipitation (IP) using an anti-FLAG antibody. IP was performed routinely using antibody-deficient samples as negative controls. In IP without antibody, no signal corresponding to NCC can be detected with an anti-NCC antibody. Each lane represents a different sample.

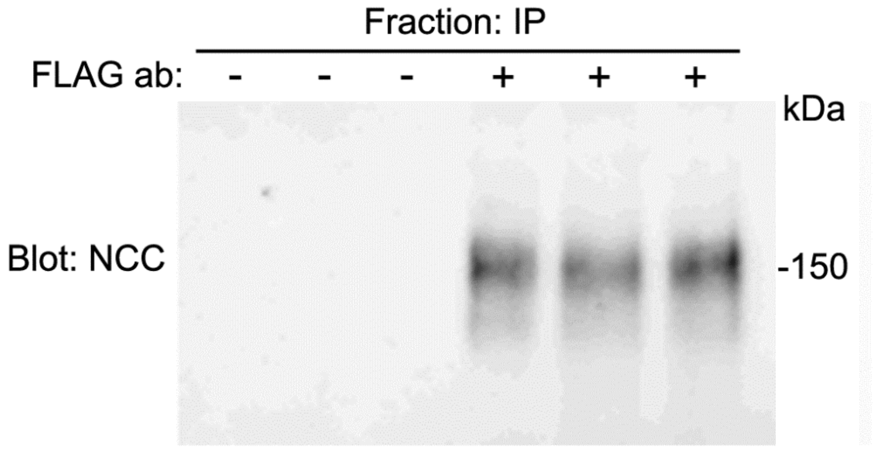

Supplemental Figure 2. Specificity of the antibody targeting phosphorylated Threonine-58 NCC (pT58). Various MDCKI-rNCC cell lines with arginine/aspartic acid mutations at T58 (each lane represents a different cell line) were probed with either total NCC or a pT58-NCC specific antibody [9]. Total NCC was observed in all cell lines and whole kidney (WK) but not non-transfected control cells (NT). In contrast, signal using the pT58-NCC antibody was only observed in WK samples. Actin was used as a loading control.

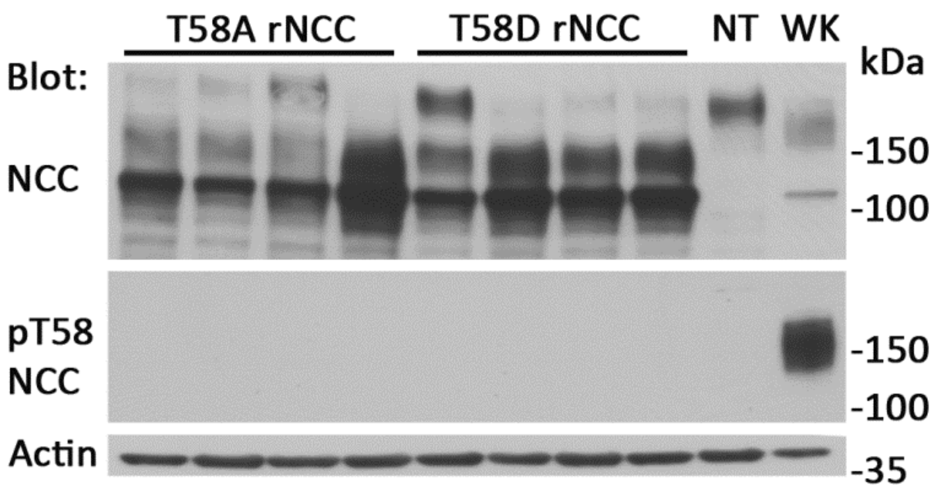

Supplement: Supplementary file 1 — Supplemental Figures and Legends [file 41598_2017_12819_MOESM1_ESM.pdf]
